# Pre-operative Circulating Plasma Gelsolin Predicts Residual Disease and Detects Early Stage Ovarian Cancer

**DOI:** 10.1038/s41598-019-50436-1

**Published:** 2019-09-26

**Authors:** Meshach Asare-Werehene, Laudine Communal, Euridice Carmona, Tien Le, Diane Provencher, Anne-Marie Mes-Masson, Benjamin K. Tsang

**Affiliations:** 1 Departments of Obstetrics & Gynecology and Cellular & Molecular Medicine, University of Ottawa, Ottawa, K1H 8L1 Ontario Canada; 2 State Key Laboratory of Quality Research in Chinese Medicine, Macau Institute for Applied Research in Medicine and Health, Macau University of Science and Technology, Avenida Wai Long, Taipa, Macao China; 3 Chronic Disease Program, Ottawa Hospital Research Institute, Ottawa, K1H 8L6 Ontario Canada; 4 Centre de recherche du CHUM et Institut du cancer de Montréal, Montréal, H2X 0A9 Québec Canada; 5 Division of Gynecologic Oncology, Department of Obstectrics and Gynecology, University of Ottawa, Ottawa, K1H 8L6 Ontario Canada; 6 Division of Gynecologic Oncology, Department of Obstectrics and Gynecology, Université de Montréal, Montréal, H3C 3J7 Québec Canada; 7 Department of Medicine, Université de Montréal, Montréal, H3C 3J7 Québec Canada

**Keywords:** Ovarian cancer, Tumour biomarkers

## Abstract

Ovarian cancer (OVCA) patients with suboptimal residual disease (RD) and advanced stages have poor survival. pGSN is an actin binding protein which protects OVCA cells from cisplatin-induced death. There is an urgent need to discover reliable biomarkers to optimize individualized treatment recommendations. 99 plasma samples with pre-determined CA125 were collected from OVCA patients and pGSN assayed using sandwich-based ELISA. Associations between CA125, pGSN and clinicopathological parameters were examined using Fisher’s exact test, T test and Kruskal Wallis Test. Univariate and multivariate Cox proportional hazard models were used to statistically analyze clinical outcomes. At 64 µg/ml, pGSN had sensitivity and specificity of 60% and 60% respectively, for the prediction of RD where as that of CA125 at 576.5 U/mL was 43.5% and 56.5% respectively. Patients with stage 1 tumor had increased levels of pre-operative pGSN compared to those with tumor stage >1 and healthy subjects (*P* = 0.005). At the value of 81 µg/mL, pGSN had a sensitivity and specificity of 75% and 78.4%, respectively for the detection of early stage OVCA. At the value of 0.133, the Indicator of Stage 1 OVCA (ISO1) provided a sensitivity of 100% at a specificity of 67% (AUC, 0.89; *P* < 0.001). In the multivariate Cox regression analysis, pGSN (HR, 2.00; CI, 0.99–4.05; *P* = 0.05) was an independent significant predictor of progression free survival (PFS) but not CA125 (HR, 0.68; CI, 0.41–1.13; *P* = 0.13). Pre-operative circulating pGSN is a favorable and independent biomarker for early disease detection, RD prediction and patients’ prognosis.

## Introduction

Ovarian cancer (OVCA) causes more death than any gynecological cancer as a result of late presentation, frequent recurrences and ultimately chemoresistance development^1^. It is estimated that around 70% of OVCA patients are diagnosed with late stage of the disease, resulting in 5-year survival rate of ~45% due predominantly to no specific signs and symptoms in the early disease phase^2–4^. This is even more challenging in high grade serous (HGS) OVCA patients who have the worst survival outcomes compared to other histologic subtypes^2–4^. Despite considerable efforts to screen for OVCA, minimal progress has been made to date^5,6^. Although CA-125 and other circulating markers (such as CA-153, LPA, prostasin, HE4, osteopontin, kallikreins, bikunin and VEGF) have been studied, little diagnostic success has been achieved^7^. In order to improve the pre-operative diagnosis of early OVCA and recurrence, multivariate index assays (ROMA^8^, Overa^9^ and Ova 1^10^) have been developed. These assays are modestly helpful, although their significance is mostly realised in OVCA surveillance and treatment monitoring. Plasma nucleic acids, including miR-21, miR-141, miR-214, let-7b, have shown promise; however, further validation studies are needed^11–13^.

CA-125 is elevated in only ~40% of early-stage OVCA patients but more frequently elevated in advanced stages, thus making it an unsuitable candidate for early detection of the disease^5,6^. Regardless, CA125 and trans-vaginal ultrasound are widely used as screening tests for OVCA^11,14^. There are urgent needs for novel cost-effective diagnostic markers to better diagnose and manage OVCA patients. Another prognostic factor that reflects the survival of OVCA patients is the amount of residual disease (RD) after surgery^15–17^. Currently, there is no established marker to reliably predict RD pre-operatively^7^. Serum CA-125 remains a widely used marker to screen for post-treatment tumor progression^18^. However, CA-125 is less sensitive and not specific in the detection of early stage disease^11,19–21^.

pGSN is an actin binding protein and is present in the human plasma^22–25^. We have previously demonstrated that the over-expression of gelsolin protects ovarian cancer cells against cisplatin-induced death and could be a potential target for treatment^26^. pGSN has also been implicated in pathological disease conditions such as prostate cancer, breast cancer, sepsis, arthritis, microbial infections, hemodialysis and other inflammatory disorders^22,27–31^. Although pGSN is highly secreted in OVCA cells, its role in patients’ plasma has not been investigated yet. Our previous observation of the possible involvement of pGSN in chemoresistance in OVCA has prompted us to investigate its importance in the plasma of OVCA patients. In this study, we determined if pre-operative circulating pGSN is a better indicator of early stage OVCA and predictor of RD compared to CA125.

## Results

### Characteristics of patients

The validity of tumor stage was performed by certified Gynecologic-Oncology team. Clinicopathologic characteristics of the ovarian cancer patients are represented in Supplementary Table S1. Patients are classified as FIGO stages 1 (*N* = 10), 2 (*N* = 11), 3 (*N* = 67) and 4 (*N* = 11). No neoadjuvant chemotherapy or radiotherapy was used. The age of the patients ranged from 36–82 years and was dichotomized by their median into ≤61 (*N* = 51) and >61 (*N* = 48). Optimal cytoreduction was achieved in 50 patients in the cohort. CA125 and pGSN levels were measured and dichotomized by their medians (576.5 U/mL and 79 µg/mL) into low and high groups respectively.

### An elevated level of pre-operative circulating pGSN is highly associated with increased residual disease (RD)

**Figure 1:**
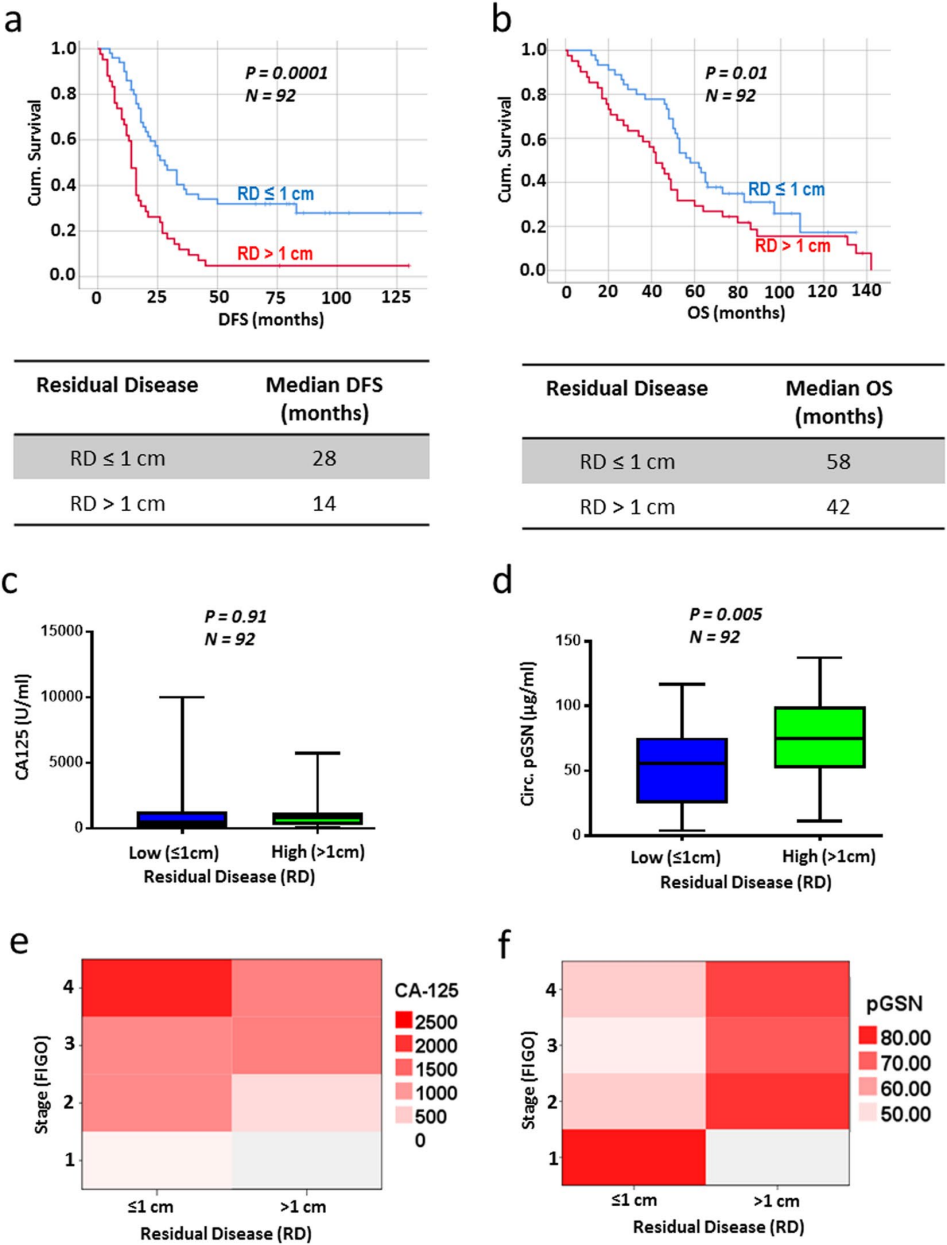
OVCA patients with high RD have significantly higher levels of circulating pGSN. (**a**,**b**) Kaplan Meier survival analysis stratified by RD (≤1 cm or >1 cm). Patients with RD ≤ 1 cm have better DFS (**a**) and OS (**b**) compared with those with RD > 1 cm. P-values were calculated by the log-rank test. Distribution of CA-125 (**c**) and pGSN (**d**) in patients with low RD (≤1 cm) and high RD (>1 cm) by box plots. Although there was no significant difference with CA-125 (**c**), high RD patients have significantly higher levels of pGSN compared with the low RD patients. Heat map was used to stratify the levels of CA-125 (**e**) and pGSN (**f**) against RD and stage. *P*-values were calculated by independent sample *t*-test.

RD is a well validated predictor of patient prognosis and survival^15,16,32^; however, there is no test to reliably predict complete, optimal or suboptimal cytoreduction pre-operatively^7^. Using a Kaplan Meier survival analysis, our patients with ≤1 cm residual disease had better DFS (Fig. 1a; *P* = 0.0001; *N* = 92; median DFS, 28 months) and OS (Fig. 1b; *P* = 0.01; *N* = 92; median OS, 58 months) compared with the >1 cm group (median DFS, 14 months; median OS, 42 months). Also, using box plots, we compared the means of CA125 and pGSN in both RD  groups and statistical significance calculated by independent sample *t*-test. Although no significant difference was observed in the levels of CA125 between both groups (Fig. 1c; *P* = ns; *N* = 92), patients with suboptimal surgical debulking had increased levels of pre-operative pGSN compared with the optimal cytoreduction group (Fig. 1d; *P* = 0.005; *N* = 92). This phenomenon was also observed when heat maps were used to stratify the means of pGSN and CA125 levels against RD (Figs. 1e,f). In Supplementary Table S2, levels of pGSN and CA125 were correlated with RD using the Pearson’s correlation test. There was a significant positive correlation between pGSN and RD (R = 0.29, *P* < 0.01, *N* = 92); however, no significant correlation was observed with CA125 (R = −0.01, *P* = 0.91, *N* = 92).

### A pre-operative plasma level of pGSN is a better predictive marker for residual disease (RD)

**Figure 2:**
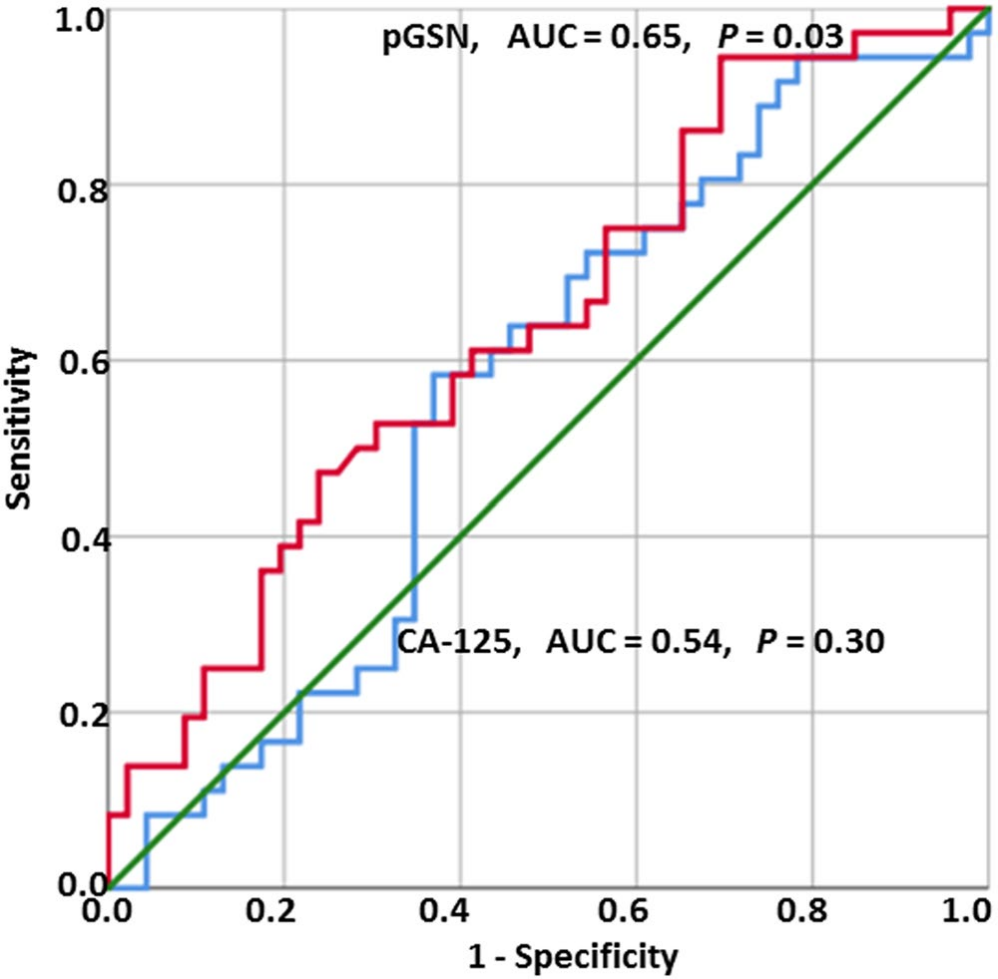
Receiving operating characteristic (ROC) curves for pGSN and CA-125 in detection of RD in OVCA patients. pGSN cutoff (64 µg/mL; sensitivity, 60%; specificity, 60%). CA125 cutoff (576.5 U/mL; sensitivity, 43.5%; specificity, 56.5%). CA-125 vs RD, *P* = 0.30; pGSN vs RD, *P* = 0.03.

To assess the performance of pGSN as a predictor of RD in OVCA patients as compared with CA125, we generated receiving operating characteristic (ROC) curves. The approximate area under the curve (AUC) derived from the ROC curve was used to assess the diagnostic performances of pGSN and CA125. The AUC of pGSN to predict suboptimal RD was 0.65 (*P* = 0.03) where as that of CA125 was 0.54 (*P* = 0.30) (Fig. 2). At the value of 64 µg/ml (optimal cut-off using Fisher’s exact test), pGSN had a sensitivity and specificity of 60% and 60% respectively, for the prediction of RD. CA125 value at 576.5 U/mL (optimal cut-off using Fisher’s exact test) had a sensitivity and specificity of 43.5% and 56.5% respectively, in predicting suboptimal RD (Supplementary Table S3).

### A pre-operative plasma level of pGSN is a significantly sensitive diagnostic tool for early stage ovarian cancer

**Figure 3:**
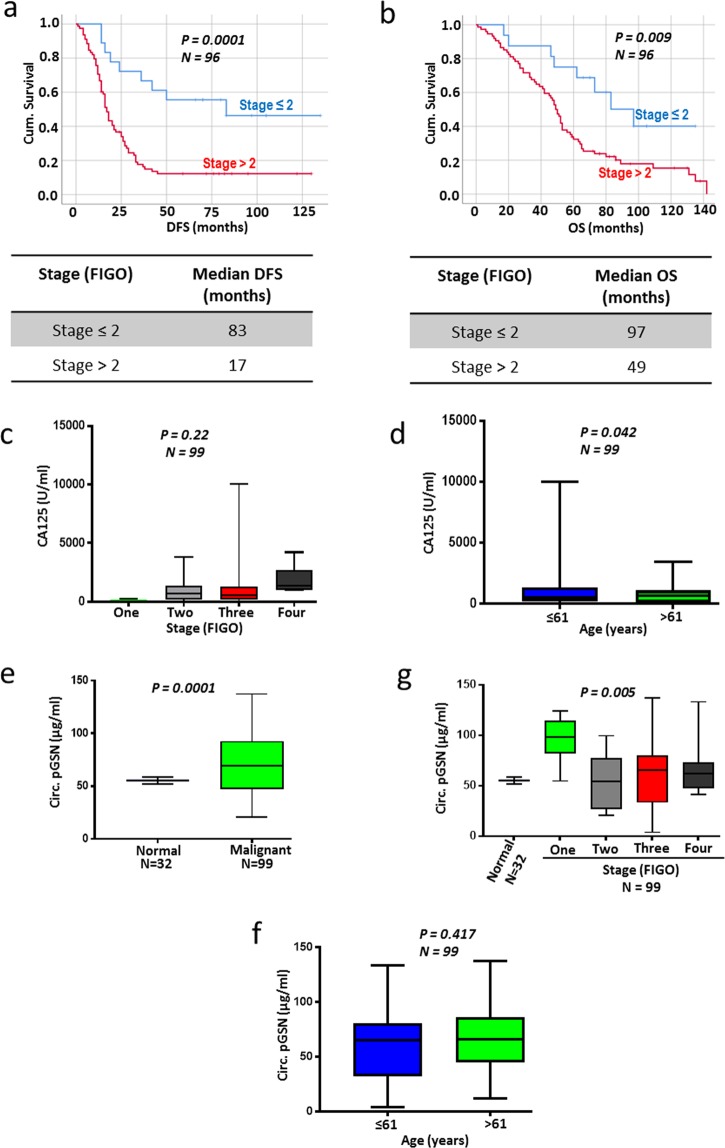
Circulating pGSN is highly detected in OVCA patients with stage 1 disease. (**a,b**) Kaplan Meier survival analysis stratified by stage showed that patients with stage ≤2 have better DFS (**a**) and OS (**b**) compared with those with stage >2. *P*-values were calculated by the log-rank test. Distribution of CA-125 (**c,d**) and pGSN (**e**–**g**) in patients stratified by stage and age using box plots. *P*-values were calculated by one-way ANOVA and independent sample *t*-test.

Patients were classified into two groups based on their tumor stage (FIGO; ≤2 and >2) and their survival were assessed using Kaplan Meier survival analysis (Figs. 3a,b). Patients with tumor stage ≤2 had better DFS (*P* = 0.0001; *N* = 96; median DFS, 83 months) and OS (*P* = 0.009; *N* = 96; median OS, 97 months) compared with their counterparts with >2 tumor stage (Fig. 3a,b; median DFS, 17 months; median OS, 49 months). Using box plots, we compared the means of CA-125 and pGSN from patients in all 4 tumor stages (FIGO; 1, 2, 3 and 4). While an increase in CA125 concentration was observed with increase in stage, this difference was not statistically significant (Fig. 3c; *P* = 0.22; *N* = 99). Patients who are more than 61 years old had decreased levels of CA125 compared with those below 61 years (Fig. 3d; *P* = 0.042; *N* = 99). pGSN levels were significantly higher in malignant patients (OVCA; *N* = 99) compared with healthy subjects (*N* = 32) without cancer (Fig. 3e; *P* = 0.0001). Patients with stage 1 tumor had abundant levels of pre-operative pGSN compared to patients with tumor stage >1 and the healthy subjects (Fig. 3f; *P* = 0.005). Age had no significant effect on the levels of pGSN in OVCA patients (Fig. 3g; *P* = 0.417; *N* = 99). The means of pGSN and CA-125 levels were stratified against tumor stage using heat maps (Figs. 1e,f).

**Figure 4:**
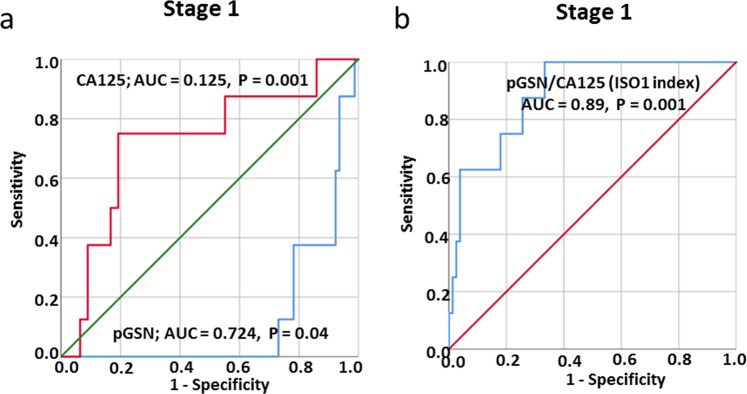
Receiving operating characteristic (ROC) curves for pGSN, CA125 and ISO1 index in the detection of stage 1 disease in OVCA patients. (**a**) pGSN cutoff (81.09 µg/mL; sensitivity, 75%; specificity, 78.4%). (**b**) ISO1 index cutoff (0.133; sensitivity, 100%; specificity, 67%). CA-125 vs stage 1, *P* = 0.001; pGSN vs stage 1, *P* = 0.04; ISO1 index vs stage 1, *P* < 0.001.

We further tested how reliable pGSN could be used as an early diagnostic tool for OVCA compared with CA-125. Using ROC curves, we derived AUC to assess the diagnostic performances of both markers. The AUC of pGSN to detect early stage OVCA was 0.724 (*P* = 0.04) where as that of CA-125 was 0.125 (*P* = 0.001) (Fig. 4a). At the value of 81 µg/mL (optimal cut-off using Fisher’s exact test), pGSN had a sensitivity and specificity of 75% and 78.4%, respectively for the detection of early stage OVCA (Supplementary Table S3). Compared with pGSN (AUC, 0.276; *P* = 0.04), CA-125 was a better marker for late stage OVCA detection with an AUC of 0.875 (*P* = 0.001) (Fig. 4a). At the value of 576.5 U/mL (optimal cut-off using Fisher’s exact test), CA-125 had a sensitivity and specificity of 55.1% and 100% respectively (Supplementary Table S3), in detecting late stage OVCA.

### Combining pGSN and CA125 in a multivariate index assay provides 100% sensitivity detection for stage 1 OVCA

To further increase the diagnostic accuracy of early stage OVCA, the pre-operative levels of pGSN and CA125 were combined in a multianalyte index assay. A formula, Indicator of Stage 1 OVCA (ISO1) index, was derived from the values of CA125 and pGSN after which the resulting values were used in a ROC curve analysis (Fig. 4b). At the value of 0.133 (optimal cut-off using Fisher’s exact test), ISO1 provided a sensitivity of 100% at a specificity of 67% (AUC, 0.89; *P* < 0.001) (Fig. 4b; Supplementary Table S3). The ISO1 index was derived as below:

$${\rm{ISO}}1\,{\rm{index}}=P/C,$$

Where

$${\rm{P}}={\rm{p}}{\rm{G}}{\rm{S}}{\rm{N}}\,({\rm{\mu }}{\rm{g}}/{\rm{m}}{\rm{L}}),\,{\rm{C}}={\rm{C}}{\rm{A}}125\,({\rm{U}}/{\rm{m}}{\rm{L}})\,{\rm{a}}{\rm{n}}{\rm{d}}\,{\rm{I}}{\rm{S}}{\rm{O}}1={\rm{I}}{\rm{n}}{\rm{d}}{\rm{i}}{\rm{c}}{\rm{a}}{\rm{t}}{\rm{o}}{\rm{r}}\,{\rm{o}}{\rm{f}}\,{\rm{S}}{\rm{t}}{\rm{a}}{\rm{g}}{\rm{e}}\,1\,{\rm{O}}{\rm{V}}{\rm{C}}{\rm{A}}.$$

### Prognostic impact of pre-operative circulating pGSN and relationship with other clinicopathologic parameter

We further evaluated the prognostic impact of pGSN, CA125 and other clinicopathological parameters using uni- and multivariate Cox regression analyses as shown in Supplementary Tables S4 and S5, respectively. Median cut-offs for pGSN and CA125 were used to predict DFS and OS. From the univariate analysis (Supplementary Table S4), FIGO stage, residual disease (RD), CA125 and pGSN showed a significant association with DFS and OS. In the multivariate Cox regression analysis (Supplementary Table S5), pGSN (HR, 2.00; CI, 0.99–4.05; *P* = 0.05) and optimal RD (HR, 0.39; CI, 0.23–0.68; *P* < 0.01) were found to be significant predictors of DFS but not CA125 (HR, 0.68; CI, 0.41–1.13; *P* = 0.13). Regarding OS, age (HR, 0.47; CI, 0.28–0.81; *P* = 0.01), RD (HR, 0.51; CI, 0.29–0.91; *P* = 0.02) and CA125 (HR, 0.47; CI, 0.27–0.82; *P* = 0.01) were found to be significantly associated with an increased risk for death.

## Discussion

In this present study, we have demonstrated for the first time that pGSN is a better diagnostic tool for the detection of early stage OVCA and prediction of suboptimal cytoreduction than the commonly used CA125. The overall sensitivity and specificity of pGSN were higher than those of CA125 in the detection of early stage OVCA. This was the same for predicting optimal RD status. The detection sensitivity for early stage OVCA was further enhanced by 35% when pGSN was combined with CA125 in a multianalyte assay analysis. CA125 however, showed a very high sensitivity and specificity for the detection of late stage OVCA. These findings support our hypothesis that circulating pGSN is a novel marker for early stage OVCA detection and optimal RD prediction.

Although much efforts are made to early detect OVCA, there is no reliable and validated circulating biomarker established^7^. Despite CA125 being used as a serum marker to detect early OVCA and monitor the clinical course of these patients, its low sensitivity presents as the greatest obstacle for its use to improve patients prognosis^7,33^. In our current study, we have demonstrated that pre-operative circulating pGSN is a better diagnostic tool than CA125 to detect early stage OVCAs. Unlike CA125, circulating pGSN levels were not affected by age hence presents as a reliable marker for diagnosis. The overall early stage detection accuracy (sensitivity; 75% and specificity; 78.4%) of pGSN was higher than that of CA125. These findings are consistent with others who have similarly shown that CA125 has a lower sensitivity in detecting early stage OVCA^34^. Although CA125 is frequently used in the clinical workup of a suspicious mass for OVCA, ~40% of all OVCA patients express little to no serum CA125 abnormalities in the early stages. This in addition to the absence of overt clinical significant symptoms leaves >70% of OVCA patients being diagnosed at the late stages of the disease^19,35,36^. Unlike CA125, circulating pGSN was significantly elevated in early stage OVCA patients compared with the late stage disease. The positive and significant correlation between pGSN levels and stage 1 OVCA could be exploited to early diagnose patients and improve their overall survival. Given the heterogeneous nature of the OVCA microenvironment and the relatively smaller HGS patients involved in this study, it is worth validating these findings in larger HGS OVCA cohorts as well as extend to other histologic subtypes such as endometroid and clear cell carcinoma.

We also have shown the combination of pGSN and CA125 in a multivariate index assay to detect early stage OVCA provided a much higher sensitivity and specificity than individual markers. Using the IS1O formula, 100% sensitivity was achieved in the detection of early stage OVCA. The IS1O formula derived from the pre-operative levels of pGSN and CA125 outperformed FDA approved OVCA multiplex assays such as ROMA (sensitivity; 89%)^33,36^ and Overa (sensitivity; 91%)^9^. Ova1 is the most recent multivariate index assay to be approved by FDA and entails two upregulated markers (CA125II, β-microglobulin) and 3 downregulated markers (apolipoprotein A1, prealbumin, transferrin) incorporated on the same detection panel^10,33^. At a specificity of 54%, Ova1 has a sensitivity of 94%^10^; a diagnostic accuracy that is below the performance of the ISO1 index. We hypothesize that the usage of the ISO1 index in combination with trans-vaginal ultrasound will further provide additional diagnostic significance to early detect OVCA patients; an intervention that will improve patient survival.

RD is considered one of the most important prognostic factors for survival in OVCA patients; however, there is no established reliable marker to predict suboptimal cytoreduction pre-operatively^15,16,32^. Although CA125 and other circulating proteins have shown promise as predictors of RD, their overall accuracy and reliability is still questionable. In this respect, there is an urgent need to identify novel predictive markers to predict RD; an effort that could improve clinical strategies and outcome in OVCA patients. In this study, we have observed for the first time that pre-operative circulating pGSN outperforms CA125 in predicting optimal RD. The sensitivity and specificity of pGSN were significantly higher compared to that of CA125; thus, presenting as a better predictor of RD. In addition to CA125, pre-operative pGSN has a higher accuracy rate to predict RD compared to other circulating markers^12,14,18–20^. This could provide significant information for counselling patients regarding surgical outcomes, influencing major decisions regarding primary cytoreduction versus neoadjuvant approach as well as explore other treatment options. In the future, it is worthwhile investigating if circulating pGSN will decline after post-cytoreduction. This could be used to determine peri-operative changes in circulating pGSN in order to predict disease-specific survival of OVCA patients.

Establishing reliable prognostic factors in OVCA is key to improving the overall management of OVCA patients. Early diagnosis, tumor grading, RD and poor performance status can affect to some extent patient survival^37^. In both uni- and multivariate analyses, pre-operative circulating pGSN also emerged in our study as an independent significant predictor – amongst all prognostic factors investigated – associated with disease free survival. This potentially provides a less invasive and inexpensive diagnostic test, which could be used together with other prognostic factors to enhance patient prognostication and optimal management. pGSN has also been evaluated in the serum of patients with disease conditions such as colon cancer, hemodialysis, arthritis, sepsis, burns and other inflammatory disorders^22^. We are the first however, to demonstrate the utility of circulating pGSN as a less-invasive prognostic marker, and more also in OVCA, where the majority of patients are diagnosed at late stages at the time of initial presentation.

We have recently shown in our *in-vitro* studies that pGSN is transported by exosomes secreted from OVCA cell lines. This has directed our research attention into differentiating the pathological significance of exosomal pGSN from the normal physiological free-circulating pGSN. We consider this as a necessary step since pGSN is one of the most abundant proteins in human plasma and hence, investigating the clinical significance of its exosomal delivery into the plasma will offer a more accurate prognostification. In addition to pre-operative sample analysis, the levels of pGSN should be further monitored in post-operative and chemotherapy/radiationtherapy/immunotherapy/targeted therapy periods to define and optimize its performance.

## Conclusion

**Figure 5:**
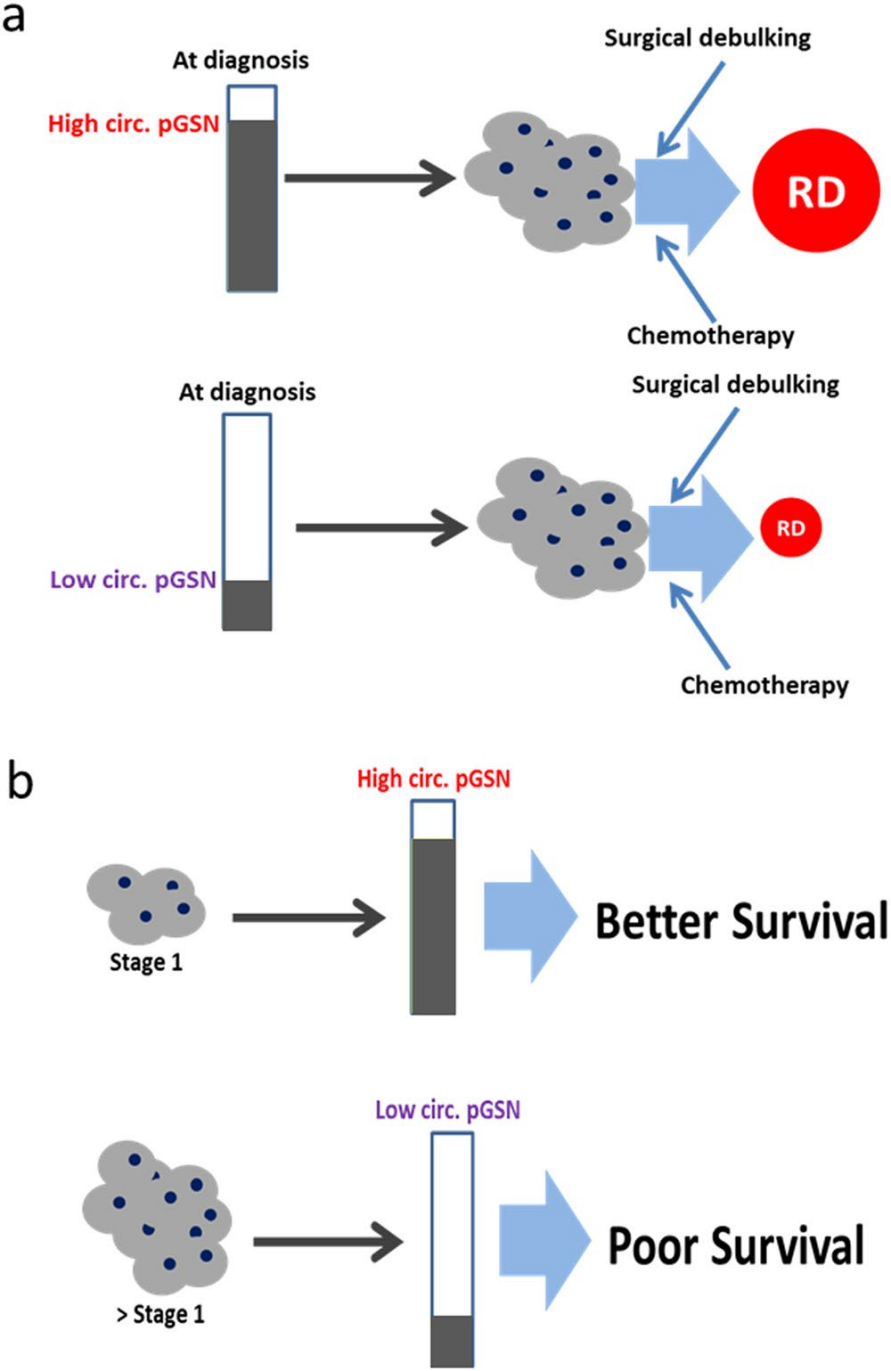
Hypothetical models illustrating how pre-treatment levels of pGSN could predict stage 1 OVCA and RD. (**a**) Increased pre-operative levels of pGSN at diagnosis could predict high RD in OVCA patients before surgery/treatment while low levels of pGSN at diagnosis could indicate minimal RD after surgery. (**b**) During the onset of OVCA (stage 1), increased levels of pGSN are detectable in the plasma of patients which could improve survival. However, in advanced stages of OVCA pGSN levels in patients’ plasma decrease, an indication of poor survival.

For the first time, we have identified in the present study pre-operative circulating pGSN as a potential marker for the detection of early stage OVCA, a predictor of optimal RD and as an independent prognostic factor in OVCA (Fig. 5); outperforming the established tumor marker, CA125. Pre-operative pGSN in the plasma could be used to discriminate early-stage OVCA patients. Pre-operative circulating pGSN is not only sensitive as an individual diagnostic marker; however, using it together with CA125 provides additional diagnostic sensitivity compared with currently FDA approved multianalyte assays. Detecting circulating pGSN by ELISA is less expensive, less labor-intensive, reproducible and commercially available, presenting as an advantage over other sophisticated techniques like whole-genome analysis, transcription profiling and proteomic analysis. While we are encouraged by the novelty and clinical importance of the present findings, we acknowledge the small population of patients involved in this study and hence involvement of patients from multiple cohorts and stratification based on histological subtypes will help not only to validate the findings observed but extent the application of the current study.

## Patients and Methods

### Data reporting

A convenient sample size was chosen. During samples preparation and analyses, plasma samples were blinded.

### Ethics statement

This study was approved by the Centre hospitalier de l’Universite de Montreal (CHUM) ethics committee (IRB approval number; BD 04-002) and the Ottawa Health Science Network Research Ethics Board (IRB approval number; OHSN-REB 1999540-01 H) and conducted in accordance with the appropriate guidelines. Written informed consent was obtained from all subjects.

### Plasma samples

99 plasma samples with predetermined CA125 levels (HGS; 69, LGS; 4, not verified; 26) were collected from ovarian cancer patients at the CHUM from 1992 to 2012. Prior to plasma collection, patients did not receive any neoadjuvant chemotherapy or radiotherapy. pGSN was measured in these samples as well as plasma samples from 32 healthy non-cancerous subjects. Details of patient population and demographics are outlined in Supplementary Table S1. All samples were examined by one gynecologic-oncologic pathologist who assigned tumor grade and histopathological subtype according to the International Federation of Gynecology and Obstetrics (FIGO) criteria. All patients were managed with primary surgery. They include optimal residual disease (RD) categorized as being ≤1 cm and suboptimal residual disease (RD) defined as being >1 cm. Disease-free (DFS) and overall (OS) survival assessment was based on CA125 and CT imaging during follow-up. DFS was calculated from time of diagnosis to time of recurrence and OS from time of diagnosis to time of death.

### ELISA

Pre-operative pGSN concentrations were assayed in OVCA patients (n = 99) by sandwich ELISA (Aviscera Bioscience, Inc. CA), according to manufacturer’s instructions. The detection antibody was raised against human plasma (soluble) gelsolin. Plasma samples were diluted in a sample buffer (1/1500; Aviscera Bioscience, Inc. CA) and all analyses done in triplicate. Optical densities (OD) were determined using a microtiter plate reader at 450 nm. The blank was subtracted from the triplicate readings for each standard and sample and concentrations reported in µg/mL.

### Biostatistical methods

The SPSS software version 25 (SPSS Inc., Chicago, IL, USA) and Graphpad Prism 7 (San Diego, CA, USA) were used to perform all statistical analyses and two-sided *P* ≤ 0.05 considered to indicate statistical significance. Receiving operating characteristic (ROC) curves were used to assess the performances of pGSN and CA125 over their entire range of values. The area under the curve (AUC) was used as an index of global test performance. pGSN and CA125 concentrations were dichotomized by their medians cut-offs into low or high and correlated to residual disease (RD) and early stage using Pearson’s correlation test (two-tailed). The means of pGSN and CA125 levels were plotted against age, stage and RD using bar chat and statistical analyses performed by using unpaired *t*-test and one-way ANOVA; Gaussian distribution was tested. The means of pGSN and CA125 levels were plotted against stage, age and RD using box plots. The box represents the interquartile (IQ) range, and the whiskers represent the highest and lowest values, which are no greater than 1.5 times the IQ range. Outliers are values greater than 1.5 times (circle) or 2 times (stars) the IQ range. The relationship of these dichotomous variables to other clinicopathologic correlates was examined using Fisher’s exact test, T test and Kruskal Wallis Test as appropriate. Survival curves (DFS and OS) were plotted with Kaplan Meier and *P*-values calculated using the log-rank test. Univariate and multivariate Cox proportional hazard models were used to assess the hazard ratio (HR) for CA125, pGSN, stage (FIGO), RD and age as well as corresponding 95% confidence intervals (CIs).

## Supplementary information

Supplementary Information
