# Supplementary material for: Pre-operative Circulating Plasma Gelsolin Predicts Residual Disease and Detects Early Stage Ovarian Cancer
Source: Sci Rep. 2019 Sep 26;9:13924. doi: 10.1038/s41598-019-50436-1 (PMC6763481; doi:10.1038/s41598-019-50436-1)
Supplement: Supplementary file 1 — Supplementary Information [file 41598_2019_50436_MOESM1_ESM.docx]

**Pre-operative Circulating Plasma Gelsolin Predicts Residual Disease and**

**Detects Early Stage Ovarian Cancer**

Meshach Asare-Werehene^a,c^, Laudine Communal^d^, Euridice Carmona^d^, Tien Le^e^, Diane Provencher^d,f^, Anne-Marie Mes-Masson^d,g^, and *Benjamin K. Tsang^a,b,c^

^a^Departments of Obstetrics & Gynecology and Cellular & Molecular Medicine, University of Ottawa, Ottawa, Ontario, Canada, K1H 8L1;

^b^State Key Laboratory of Quality Research in Chinese Medicine, Macau Institute for Applied Research in Medicine and Health, Macau University of Science and Technology, Avenida Wai Long, Taipa, Macao, China;

^c^Chronic Disease Program, Ottawa Hospital Research Institute, Ottawa, Ontario, Canada K1H 8L6

^d^Centre de recherche du CHUM et Institut du cancer de Montréal, Montréal, Québec, Canada H2X 0A9

^e^Division of Gynecologic Oncology, Department of Obstectrics and Gynecology, University of Ottawa, Ottawa, Ontario Canada K1H 8L6

^f^Division of Gynecologic Oncology, Department of Obstectrics and Gynecology, Université de Montréal, Montréal, Québec, Canada H3C 3J7

^g^Department of Medicine, Université de Montréal, Montréal, Québec, Canada H3C 3J7

***Correspondence:** Dr. Benjamin K Tsang, Chronic Disease Program, Ottawa Hospital Research Institute, The Ottawa Hospital (General Campus), Ottawa, Canada K1H 8L6; Tel: 1-613-798-5555 ext 72926; Email: [btsang@ohri.ca](mailto:btsang@ohri.ca)

| Variable | Number of Patients |
| --- | --- |
| Age (Range; 36 – 82 years)  ≤61  >61 | 51  48 |
| Stage (FIGO)  1  2  3  4 | 10  11  67  11 |
| Stage (FIGO)  ≤2  >2 | 21  78 |
| Histological Subtypes  Not verified  High Grade Serous (HGS)  Low Grade Serous (LGS) | 26  69  4 |
| Residual disease (RD)  ≤1 cm  >1 cm | 50  42 |
| CA-125 (U/ml)^a^  Low (≤576.5)  High (>576.5) | 49  50 |
| pGSN (µg/ml)^a^  Low (≤79)  High (>79) | 70  29 |
| pGSN (µg/ml)^a^  Not verified  HGS  LGS | 25  70  4 |
| Healthy Subjects | 32 |

**Table 1. Characteristics of patients**

^a^Median cut-off value.

CA-125, cancer antigen 125; pGSN, plasma gelsolin; FIGO, International Federation of Gynecology and Obstetrics.

**Table 2. Correlation between plasma pGSN and CA-125 levels and ovarian cancer residual disease and stage.**

|  | | **CA-125 (U/ml)** | **pGSN (µg/ml)** |
| --- | --- | --- | --- |
| **Residual disease (RD)** | R^a^  Sig. (2-tailed)  N^b^ | -0.01  0.91  92 | 0.29  <0.01  92 |
| **Stage (FIGO)** | R^a^  Sig. (2-tailed)  N^b^ | 0.22  0.04  99 | -0.20  0.05  99 |

^a^Pearson’s correlation, ^b^Number of patients.

CA-125, cancer antigen 125; pGSN, plasma gelsolin; FIGO, International Federation of Gynecology and Obstetrics.

**Table 3. The sensitivities and specificities of OVCA plasma biomarkers.**

| **Biomarkers** | **Sensitivity (%)** | **Specificity (%)** | **AUC^^^** |
| --- | --- | --- | --- |
| **Stage 1 OVCA**  **CA125**  **pGSN**  **ISO1^*^ Index (pGSN/CA125)** | 0  75  100 | 44.9  78.4  67 | 0.125  0.724  0.89 |
| **Residual disease**  **CA125**  **pGSN** | 43.5  60 | 56.5  60 | 0.54  0.65 |
| **Late Stage (3 and 4)**  **CA125**  **pGSN** | 55.1  21.6 | 100  0 | 0.875  0.276 |

OVCA, ovarian cancer; CA125, cancer antigen 125; pGSN, plasma gelsolin. *Indicator of Stage 1 OVCA. ^Area under the curve

**Table 4. Univariate Cox regression analysis for disease-free and overall survival**

| **Univariate** | | | | | | |
| --- | --- | --- | --- | --- | --- | --- |
| **Variable** | **DFS** | | | **OS** | | |
|  | **HR^*^** | **95% CI^^^** | ***P-*value** | **HR^*^** | **95% CI^^^** | ***P*-value** |
| **Age (years)**  **≤61 vs >61** | 0.91 | 0.58 – 1.43 | 0.68 | 0.66 | 0.41 – 1.07 | 0.91 |
| **Stage (FIGO)**  **≤2 vs >2** | 0.30 | 0.15 – 0.60 | <0.01 | 0.39 | 0.19 – 0.82 | 0.01 |
| **Stage**  **1 vs >1** | 0.15 | 0.04 – 0.60 | <0.01 | 0.23 | 0.06 – 0.93 | 0.04 |
| **RD (cm)**  **≤1 vs >1** | 0.31 | 0.19 – 0.53 | <0.01 | 1.54 | 1.13 – 2.10 | <0.01 |
| **CA-125**  **Low vs high** | 0.59 | 0.36 – 0.97 | 0.04 | 0.48 | 0.28 – 0.80 | 0.01 |
| **pGSN**  **Low vs high** | 1.81 | 1.05 – 3.12 | 0.03 | 1.94 | 1.08 – 3.48 | 0.03 |

HR, hazard ratio; DFS, disease free survival; OS, overall survival; CI, confidence interval; RD, residual disease; CA-125, cancer antigen 125; pGSN, plasma gelsolin; FIGO, International Federation of Gynecology and Obstetrics; vs, versus.

^*^Estimated from Cox proportional hazard regression model.

^^^Confidence interval of the estimated HR.

**Table 5. Multivariate Cox regression analysis for disease-free and overall survival**

| **Multivariate Analysis** | | | | | | |
| --- | --- | --- | --- | --- | --- | --- |
| **Variable** | **DFS** | | | **OS** | | |
|  | **HR^*^** | **95% CI^^^** | ***P*-value** | **HR^*^** | **95% CI^^^** | ***P*-value** |
| **Age (years)**  **≤61 vs >61** | 0.76 | 0.47 – 1.25 | 0.29 | 0.50 | 0.29 – 0.86 | 0.01 |
| **Stage (FIGO)**  **≤2 vs >2** | 0.63 | 0.27 – 1.47 | 0.29 | 0.63 | 0.25 – 1.57 | 0.32 |
| **Stage**  **1 vs >1** | 0.40 | 0.08 – 2.00 | 0.27 | 0.78 | 0.15 – 4.04 | 0.76 |
| **RD (cm)**  **≤1 vs >1** | 0.40 | 0.23 – 0.68 | <0.01 | 0.51 | 0.28 – 0.91 | 0.02 |
| **CA-125**  **Low vs high** | 0.68 | 0.41 – 1.13 | 0.13 | 0.47 | 0.27 – 0.82 | 0.01 |
| **pGSN**  **Low vs high** | 2.00 | 0.99 – 4.05 | 0.05 | 2.10 | 0.94 – 4.33 | 0.07 |

HR, hazard ratio; DFS, disease free survival; OS, overall survival; CI, confidence interval; RD, residual disease; CA-125, cancer antigen 125; pGSN, plasma gelsolin; FIGO, International Federation of Gynecology and Obstetrics; vs, versus.

^*^Estimated from Cox proportional hazard regression model.

^^^Confidence interval of the estimated HR.
